# Risk Factors for Ocular Infection with *Chlamydia trachomatis* in Children 6 Months following Mass Treatment in Tanzania

**DOI:** 10.1371/journal.pntd.0000978

**Published:** 2011-03-15

**Authors:** Luis Carlos Cajas-Monson, Harran Mkocha, Beatriz Muñoz, Thomas C. Quinn, Charlotte A. Gaydos, Sheila K. West

**Affiliations:** 1 Dana Center for Preventive Ophthalmology, Wilmer Eye Institute, Johns Hopkins University, Baltimore, Maryland, United States of America; 2 Kongwa Trachoma Project, Kongwa, Tanzania; 3 International Chlamydia Laboratory, Department of Infectious Diseases, Johns Hopkins University, Baltimore, Maryland, United States of America; 4 Division of Intramural Research, National Institute of Allergy and Infectious Diseases, National Institutes of Health, Bethesda, Maryland, United States of America; University of Cambridge, United Kingdom

## Abstract

**Background:**

Trachoma is the leading infectious cause of blindness in the world, and for endemic communities, mass treatment with azithromycin reduces the pool of infection. High coverage is essential, especially in children as they are the infectious reservoir. However, infection remains post-mass treatment. We sought to determine risk factors for infection in children post-mass treatment.

**Methodology:**

All children under 9 years in 4 villages in Tanzania were followed from baseline pre-mass treatment to six months post treatment. 1,991 children under nine years were enrolled in the longitudinal study and data on individual and household characteristics was collected at baseline. Clinical trachoma was determined by an ocular exam and infection detected by PCR of an eyelid swab. Azithromycin was offered and infection was reassessed at 6 months. A multilevel logistic regression model was used, accounting for household clustering of children for analysis.

**Principal Findings:**

Baseline infection was 23.7% and at 6 months was 10.4%, despite 95% coverage. Infection at baseline was positively associated with infection at 6 months (OR = 3.31, 95%CI 2.40–4.56) and treatment had a protective effect (OR = 0.45, 95%CI 0.25–0.80). The age group 2–4 years had an increased risk of infection at 6 months. The household characteristics predictive of infection at 6 months were increasing number of children infected in the household at baseline and increasing number of untreated children in the household.

**Conclusions:**

While one round of mass treatment with high coverage did decrease infection by over 50%, it appears that it is not sufficient to eliminate infection. Findings that young children (ages 2–4 years) and households with increasing numbers of infected and untreated children have a positive association with infection at 6 months suggest that such households could be targeted for more intensive follow up.

## Author Summary

Trachoma control programs aim for high coverage of endemic communities with oral azithromycin to reduce the pool of infection with *Chlamydia trachomatis*. However, even with high coverage, infection is seen following treatment. In four communities in Tanzania, we followed every child aged under ten years from baseline through treatment to six months post-treatment. We determined who had infection at baseline and who still had or developed infection six months later. Coverage was over 95% in children in these communities, and infection in these children decreased by over 50% at six months. The study found that, at baseline, uninfected children who were treated had prevalence of infection at 6 months of 6%, but infected children who were treated had prevalence of infection of 22% at 6 months. Other risk factors for infection at 6 months included living in a household with other infected children, and living in a household with untreated children. Our data suggest that households with untreated children might be targeted for more intensive follow up to increase coverage and reduce subsequent infection in the community.

## Introduction

Trachoma is a blinding chronic conjunctivitis caused by repeated episodes of ocular *Chlamydia trachomatis* infection. It continues to be the leading infectious cause of blindness in the world, largely in the most resource poor countries [1]. The World Health Organization (WHO) has made a commitment to eliminate blinding trachoma by the year 2020 (Global Elimination of Blinding Trachoma) [2]. The strategy designed for this initiative can be summarized with the acronym, SAFE, Surgery for trichiasis, Antibiotic to reduce the community pool of *C. trachomatis* infection, Facial cleanliness, and Environmental change to reduce transmission.

In 1999, a study by Schachter et al. demonstrated that azithromycin could be effective in a mass treatment campaign to reduce the infection in endemic villages. The manufacturer has since provided the drug free of charge to trachoma endemic countries that apply for donation [3]. Eliminating infection with a single dose of antibiotic is very appealing, because of improved compliance. In theory, infection could potentially be eliminated from a population in a single effort. This has been reported in some villages that have low trachoma prevalence [4].

However, in hyper-endemic villages, infection is still found in treated children following mass treatment. The biggest reservoir of active disease and infection is seen in children [5]. Two months after mass treatment of a hyper-endemic village in Tanzania, more than 30% of children with high bacterial loads at baseline continued to be infected [6]. In hyper-endemic villages, where active trachoma may be as high as 50% in children in cross sectional surveys, it has been suggested that up to 6 rounds of mass treatment may be necessary to eliminate infection [7].

In order to further investigate infection following mass treatment, we sought to determine the household and child factors that predict infection post treatment. Identifying such factors could help determine how to implement mass treatment.

## Methods

### Procedures

At the beginning of 2009 a household census was performed in 4 villages in Kongwa district, Tanzania. Children less than 9 years of age were identified and their parents were invited to have their children participate in a longitudinal study of trachoma and infection over a three year period. Written informed consent was obtained from all parents or guardians of children in this study. All procedures for the study were approved by the Johns Hopkins University Institutional Review Board (JHU IRB) and the National Institute of Medical Research (NIMR) in Tanzania.

Data on the potential risk factors at the individual and household levels were collected at baseline. The individual characteristics included age, gender, active trachoma and presence of *Chlamydia trachomatis* at baseline, and facial cleanliness in children 5 years of age and younger. The household characteristics were: distance to the nearest source of water (measured as self report of the time to walk one way), presence of a latrine as observed by the interviewer at the household, and average years of education of the head of the household based on self report. Facial cleanliness was assessed on children ages five years and younger by direct observation, using three elements: presence of ocular or nasal crusting, and observation of one or more flies on the face in a three second window, as measured at the time of the exam [8].

Active trachoma and presence of *C. trachomatis* were also determined at 6 months.

Trained trachoma graders performed an ocular exam using 2.5x loupes and a torch to determine active trachoma. The trachoma graders were standardized during a two week training exercise prior to the start of the study. Inter-observer agreement, and agreement with a senior grader had to be above kappa = 0.6 for TF and TI. Graders are monitored every six months by comparison of grades against the senior grader using a series of trachoma images [9]. Active trachoma was graded using the World Health Organization simple grading scheme [10].

An eyelid-conjunctival swab was obtained to assess presence of *C. trachomatis* DNA. These swabs were taken using strict protocols to avoid contamination. Both the trachoma grader and eyelid flipper used gloves and changed them between each child examined. In addition, “air control” swabs were taken on a random sample of 5% of children. The swabs were waved in the air above the child and not touched to the eyelid, marked as per any other sample and sent for processing. If positive, further investigation were carried out to determine the source of contamination. The samples were kept at −20°C until they were sent to the International Chlamydia Laboratory at Johns Hopkins University for processing. The samples were analyzed using real-time Polymerase Chain Reaction (Amplicor: Roche Molecular Systems, Indianapolis, IN) according to manufacturer's directions. Optical density was used to identify positive results and defined as >0.7. Optical densities between 0.2 and 0.7 were considered equivocal and were retested. Dummy swabs were performed to test for contamination in the field and laboratory. Results of dummy swabs showed evidence of lab contamination that affected 34 samples at baseline. Contaminated samples were excluded; all further tests for contamination were negative at baseline and follow up.

A single dose of azithromycin at a dose of 20 mg/kg up to 1 g was offered to all residents 6 months of age and older in each of the 4 villages by community treatment assistants, who observed and recorded treatment. Infants under 6 months of age were treated using topical tetracycline (6 weeks of 1% tetracycline topical ointment twice daily). Treatment was immediately after the time of the baseline assessment.

### Statistical Analysis

Contingency table analysis was used to identify factors associated with infection at six months, corresponding p-values were obtained from a logistic model with infection at 6 months as the outcome, accounting for clustering at the household level. In the multivariate stage, logistic regression was used to model presence of infection at six months as a function of the factors found to be associated at a 0.15 level in the univariate analysis, a backward elimination strategy was used to find a parsimonious model. Among variables whose p-value was greater than 0.05, we deleted the one that had the highest p-value. We proceeded iteratively until all variables in the model had associated p-values less than 0.05. The generalized estimating equation (GEE) approach was used to correct the standard errors to account for the correlation among children members of the same house (procedure GENMOD in SAS, binomial distribution, logit link function, exchangeable correlation structure).

## Results

**Figure 1:**
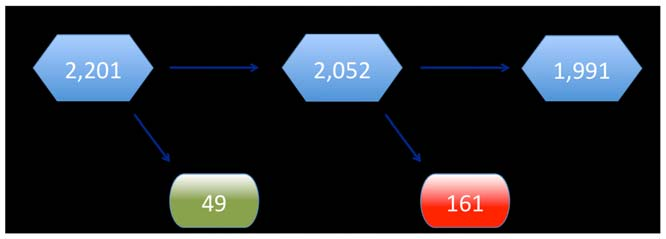
Sample population.

There were a total of 2201 children less than 9 years of age in the four villages at the time of census (Figure 1). A total of 49 (2.2%) of the children did not participate in the study at baseline. An additional 161 (7.5%) did not return at 6 months for the ocular exam. Thus, our study population consisted of 1991 children with collected data at baseline and 6 months.

A comparison of baseline characteristics of the study population and the 161 children who missed the 6-month ocular exam revealed no significant differences for all characteristics except treatment (Table 1). The study population had antibiotic coverage of 95.0% while the children with missing 6 month data had 87.5% coverage, reflecting the fact that some of them left the study even prior to mass treatment. Of note, the baseline prevalence of infection in study children was 24% compared to 22% in those who did not participate at 6 months, a non-significant difference. Overall the prevalence of Chlamydia infection and active trachoma respectively were 23.7% and 27.8% in the study population at baseline.

**Table 1:** Baseline characteristics of the 1991 children in the study sample vs. 161 children who had no data at 6 months.

	Missing at 6 Months	Study Sample	P**
% Active Trachoma	27.3	27.8	0.9
% PCR Infection*	21.7	23.7	0.57
% Unclean Faces	51.3	51.7	0.9
Age (mean)	3.9	4.0	0.54
% Female	48.4	50.9	0.55
% Treated	87.5	95.0	0.0001

*34 children had unusable PCR at baseline.

**chi square tests and a t-test for difference in mean ages.

At 6 months, 203 children were infected (10.4%), down from 23.7% at baseline. Characteristics associated with infection at 6 months are shown in table 2. There was no difference in infection at 6 months by gender or those with clean or unclean faces at baseline or the available household characteristics (education, distance to water, presence of a latrine). Prevalence of infection at 6 months were higher in those 2–4 years of age, with baseline infection, and in the untreated group (Table 2). Children were more likely to be infected at 6 months if other children in the household were infected at baseline (7%, 15%, 28% for 0, 1, 2+ infected children, test for trend p = 0.001). In addition the risk of infection increased with the number of untreated children in the same household (test for trend p = 0.02).

**Table 2:** Baseline Characteristics of the Cohort and percentage infected at 6 months.

Baseline Characteristics	N	% Infected at 6 Months	p-value &
Gender			
Male	978	9.81	0.42
Female	1012	11.0	
Age			
<2	473	9.09	REF
2–4	652	14.1	0.006
5–9	865	8.32	0.60
Infection*			
Yes	463	23.1	<0.0001
No	1493	6.43	
Treated			
Yes	1891	9.78	0.002
No	99	22.2	
Child age 0–5 with clean face at baseline**			
Yes	644	10.1	0.10
No	690	12.9	
Number of infected children in the household at baseline***:			
0	1395	7.2	
1	334	15.3	<0.001
2	146	27.7	
3+	81	28.4	
Number of untreated children in the household***			
0	1858	9.6	0.02#
1	98	19.4	
2+	36	25.0	
Other Children with TF and/or TI in HH at baseline***			
Yes	403	14.9	0.002
No	1587	9.3	
Resident of a household>60 minutes from water source			
Yes	1486	11.1	0.15
No	504	8.3	
Resident a household with Latrine			
Yes	1336	10.9	0.38
No	654	9.3	
Education of head of household ****			
None	1044	10.9	REF
Less than primary (1–6)	151	6.6	0.11
Primary or more (7–12)	791	10.4	0.70

*34 specimens were unusable at baseline

**Only children in this age group had assessment of facial cleanliness at baseline.

***the number reflects other children in household infected/having clinical disease/not treated, not counting the index child

****4 households missing information

&From a logistic model with infection at 6 months as the outcome accounting for clustering at the household level,

#linear trend.

The effect of treatment seemed to be different depending on the infection status at baseline (Table 3). Among infected children, children who were treated at baseline had 6–month prevalence of infection of 22.0%, compared to 5.9% in uninfected children who were treated. Children who were untreated and infected at baseline had a 3.1-fold higher chance of being infected at 6 months compared to untreated and uninfected children, 50.0% vs. 16.0%. However, after adjusting for other factors the interaction between infection at baseline and the effect of treatment was not statistically significant (p = 0.59).

**Table 3:** Interaction of baseline infection and treatment, and infection at 6 months**.

	N	% infected at 6 months	P***
Not Infected at baseline*			
Treated at baseline	1,412	5.9	0.001
Not treated at baseline	81	16.0	
Infected at baseline*			
Treated at baseline	445	22.0	0.09
Not treated at baseline	18	50.0	

*34 specimens were unusable at baseline.

**Interaction of treatment and baseline infection status, adjusted for age and clustering at household level, was not significant, p = .59.

***From a logistic model with infection at 6 months as the outcome accounting for clustering at the household level.

Of the sub group of 1493 children without infection at baseline, 6.4% were infected at 6 months. In this sub group, treatment was the only explanatory factor comparing those who stayed free of infection with those 96 who developed infection at 6 months. Among those free of infection, 95.1% had been treated compared to 86.4% of those who developed infection. We found no difference in gender, mean age, percentage of children under 5 years of age with unclean faces, or whether at baseline there was another infected child in the house (Table 4).

**Table 4:** Baseline characteristics of the sub group of 1439 children who were not infected at baseline, and infected at 6 months (n = 96).

Baseline Characteristics	N	% Infected at 6 Months	P***
Gender			
Male	738	6.50	0.91
Female	755	6.36	
Age			
<2	392	6.63	REF
2–4	464	7.32	0.68
5–9	637	5.65	0.53
Treated			
Yes	1412	5.88	0.003
No	81	16.0	
Child age 0–5 with clean face at baseline *			
Yes	495	6.67	0.99
No	498	6.62	
Other Children infected in household at baseline:**			
Yes	261	7.66	0.45
No	1232	6.17	

*Only children in this age group had assessment of facial cleanliness at baseline.

**The number reflects other children in household infected, not counting the index child.

***From a logistic model with infection at 6 months as the outcome accounting for clustering at the household level.

Our final model predicted infection at 6 months, adjusted for multiple variables and household clustering (Table 5). Factors that independently increased the odds of having infection at 6 months included being in the age group 2–4 years old (OR = 1.41), having infection at baseline (OR = 3.31), and living in a household with other infected children (OR = 1.39 per additional infected child). Treatment significantly reduced the odds of infection at 6 months (OR = 0.45), and other untreated children in the household were associated with increased risk (OR = 1.58 per additional untreated child).

**Table 5:** Multivariate logistic regression model predicting infection at 6 months*.

Characteristic	OR	95% Confidence Interval
Age		
<2 yrs	Ref	
2–4	1.41	1.00–2.00
5–9	0.78	0.54–1.13
Treated at baseline:		
No	Ref	
Yes	0.45	0.25–0.80
Infected at baseline		
No	Ref	
Yes	3.31	2.40–4.56
Number of Infected Children in household (per child)	1.39	1.18–1.64
Number of untreated children in household (per child)	1.58	1.08–2.31

*adjusted for clustering of children in the households.

## Discussion

In these four endemic communities we found an overall prevalence of Chlamydia infection at baseline of 23.7%. Mass antibiotic treatment coverage reached 95% of the children in the population, which is close to a goal of full coverage of all children. Despite such high coverage, the infection at 6 months was 10.4%.

One factor that predicted infection at 6 months was infection at baseline. This is consistent with a similar finding in a study from another village with similar endemicity in Tanzania [6] [11]. At six months, these infections may be due to lack of treatment, although with high coverage these are few case; the infections may also be due to high bacterial loads in those treated, which made them less likely to be cured after a single dose. West et al found previously that those children with the highest bacterial load pre-treatment were most likely to still have infection 2 months after treatment [6]. Potentially, the dose of azithromycin could be increased for these children, but a randomized trial of 30 mg/kg versus 20 mg/kg dose in children with severe trachoma did not demonstrate any difference in outcome [12]. Recent data from Ethiopia suggests that more frequent treatment, perhaps every six months, would be more effective, although this needs to be confirmed [7]. The feasibility for programs of implementing treatment even every six months to families of young children would need careful study of the cost-effectiveness of such a strategy, as it would likely double the yearly costs of mass treatment as well as demand for azithromycin.

Another factor associated with infection at 6 months, independent of self-infection at baseline, was the number of children infected in the household at baseline, and number of untreated children in the household. This finding is not unexpected, as some of the infected siblings may well be the source of reinfection [13]. To the extent that not all children are treated in the household leaves a risk for other members, as intra-household transmission appears to be relatively fast [11], [13]. Another study has shown that missing treatment does not occur at random, but clusters in households, suggesting that finding households that are non participants in mass treatment, and determining strategies to improve coverage in that group may be needed 14.

Finally, the 2–4 year age group was at increased risk of infection post-mass treatment, independently of treatment status or infection status. We did not collect data on bacterial load and it is possible that this particular age group has the highest load of infection, thus most likely to remain infected post treatment. We expected the highest prevalence of infection at 6 months would be the infants who had to be treated with topical tetracycline, and for whom compliance with a six-week course could not be assured. However, infection post treatment was greatest in the 2–4 year olds, and there was no association of 6 month infection with having an infant or infected infant in the household.

One limitation to our study is that we were not able to measure chlamydial load. Thus, we cannot be certain if this is the reason that the children ages 2–4 had higher odds of infection at 6 months. Another potential limitation is loss to follow-up of children in the study. There are two points at which we lost subjects from our original eligible population. The first was at the start of the study, where 49 (2.1%) of children did not participate. This is a small number compared to the total size of the population and is unlikely to bias our results. The second point was at the 6-month follow-up visit, where 161 (7.3%) children did not return for a follow up exam. There were no differences in baseline characteristics between the 1991 children in the study sample and the 161 children missing 6-month data, except for the percentage receiving antibiotics. Thus, they may have been more likely to have infection at 6 months, but this would not be expected to bias our findings as they were no more likely to have infection at baseline. In addition, 34 of the 1991 samples (1.7%) at baseline could not be used. It is unlikely that this loss contributed to bias as the number lost was so small, and was the result of error in the laboratory, which is masked to any clinical or treatment data.

In summary, although we found that village coverage with azithromycin decreased infection at 6 months in children ages 9 and under, it did not eliminate the pool of infection. Clearly, one round of mass treatment in these communities with infection above 20% is not enough. Based on these findings, which support our previous findings in a single community, mass treatment of communities should be continued as part of the SAFE strategy to eliminate blinding trachoma. The World Health Organization recommends at least three annual mass treatment interventions as part of SAFE, and our study will provide data in the future on potential re-emergence following a second and third round of mass treatment in line with those recommendations 15.
